# The Evolution and Transmission Dynamics of Multidrug-Resistant Tuberculosis in an Isolated High-Plateau Population of Tibet, China

**DOI:** 10.1128/spectrum.03991-22

**Published:** 2023-03-13

**Authors:** Qi Jiang, Hai-Can Liu, Qing-Yun Liu, Jody E. Phelan, Feng-Xi Tao, Xiu-Qin Zhao, Jian Wang, Judith R. Glynn, Howard E. Takiff, Taane G. Clark, Kang-Lin Wan, Qian Gao

**Affiliations:** a Department of Epidemiology and Biostatistics, School of Public Health, Wuhan University, Wuhan, China; b State Key Laboratory for Infectious Disease Prevention and Control and National Institute for Communicable Disease Control and Prevention, Chinese Center for Disease Control and Prevention, Beijing, China; c Department of Immunology and Infectious Diseases, Harvard T. H. Chan School of Public Health, Boston, Massachusetts, USA; d Department of Infection Biology, London School of Hygiene & Tropical Medicine, London, United Kingdom; e Tibet Center for Disease Control and Prevention, Lhasa, Tibet Autonomous Region, China; f Department of Infectious Disease Epidemiology, London School of Hygiene & Tropical Medicine, London, United Kingdom; g Laboratorio de Genética Molecular, CMBC, IVIC, Caracas, Venezuela; h National Clinical Research Center for Infectious Diseases, The Third People’s Hospital of Shenzhen, Shenzhen, Guangdong, China; AP-HP

**Keywords:** tuberculosis, multidrug-resistant tuberculosis (MDR-TB), whole-genome sequencing (WGS), drug resistance, evolution

## Abstract

On the Tibetan Plateau, most tuberculosis is caused by indigenous Mycobacterium tuberculosis strains with a monophyletic structure and high-level drug resistance. This study investigated the emergence, evolution, and transmission dynamics of multidrug-resistant tuberculosis (MDR-TB) in Tibet. The whole-genome sequences of 576 clinical strains from Tibet were analyzed with the TB-profiler tool to identify drug-resistance mutations. The evolution of the drug resistance was then inferred based on maximum-likelihood phylogeny and dated trees that traced the serial acquisition of mutations conferring resistance to different drugs. Among the 576 clinical M. tuberculosis strains, 346 (60.1%) carried at least 1 resistance-conferring mutation and 231 (40.1%) were MDR-TB. Using a pairwise distance of 50 single nucleotide polymorphisms (SNPs), most strains (89.9%, 518/576) were phylogenetically separated into 50 long-term transmission clusters. Eleven large drug-resistant clusters contained 76.1% (176/231) of the local multidrug-resistant strains. A total of 85.2% of the isoniazid-resistant strains were highly transmitted with an average of 6.6 cases per cluster, of which most shared the mutation KatG Ser315Thr. A lower proportion (71.6%) of multidrug-resistant strains were transmitted, with an average cluster size of 2.9 cases. The isoniazid-resistant clusters appear to have undergone substantial bacterial population growth in the 1970s to 1990s and then subsequently accumulated multiple rifampicin-resistance mutations and caused the current local MDR-TB burden. These findings highlight the importance of detecting and curing isoniazid-resistant strains to prevent the emergence of endemic MDR-TB.

**IMPORTANCE** Emerging isoniazid resistance in the 1970s allowed M. tuberculosis strains to spread and form into large multidrug-resistant tuberculosis clusters in the isolated plateau of Tibet, China. The epidemic was driven by the high risk of transmission as well as the potential of acquiring further drug resistance from isoniazid-resistant strains. Eleven large drug-resistant clusters consisted of the majority of local multidrug-resistant cases. Among the clusters, isoniazid resistance overwhelmingly evolved before all the other resistance types. A large bacterial population growth of isoniazid-resistant clusters occurred between 1970s and 1990s, which subsequently accumulated rifampicin-resistance-conferring mutations in parallel and accounted for the local multidrug-resistant tuberculosis burden. The results of our study indicate that it may be possible to restrict MDR-TB evolution and dissemination by prioritizing screening for isoniazid (INH)-resistant TB strains before they become MDR-TB and by adopting measures that can limit their transmission.

## INTRODUCTION

Drug resistance in Mycobacterium tuberculosis represents a major challenge for tuberculosis (TB) control programs. In 2020, there were about half a million new rifampicin (RIF)-resistant TB cases globally, of which 78% were also resistant to isoniazid (INH) and therefore to multidrug-resistant TB (MDR-TB) (1). In 2019, China had the second-largest burden of global MDR-TB (14%), exceeded only by India (27%). China has approximately 65,000 cases of MDR-TB annually, with high rates among both newly diagnosed (7.1%) and retreated (23%) patients (1). Tibet, also known as Xizang, is a provincial autonomous region of China that has greater burdens of both TB and MDR-TB than other areas of China. In 2014, the estimated prevalence of TB in Tibet was 758/100,000 population (2), and in the provincial capital, Lhasa, 21% of new and 57% of retreated cases had MDR-TB (3).

Tibet is relatively isolated and historically has had limited population exchange with other areas of China, and our recent study showed that local selection shaped the predominant clades of M. tuberculosis circulating on the Tibetan Plateau (4). Long-term transmission and outbreaks caused by drug-resistant TB strains have been reported in Russia (5), South Africa (6), and other countries but have not yet been described in China. While concern has generally concentrated on the increased risk of MDR-TB transmission, a recent study addressed the expansion potential of strains before they develop resistance to second-line drugs (7). To limit the emergence of MDR-TB outbreak strains, it may be useful to analyze the process through which M. tuberculosis evolves resistance to multiple drugs and to clarify the transmission risk during this process.

To trace the evolution of drug resistance and investigate the factors responsible for the high burden of MDR-TB in Tibet, we analyzed the whole-genome sequences of drug-resistant M. tuberculosis to identify the mutations conferring resistance to antituberculosis drugs and to reconstruct the evolution of drug resistance in Tibet based on the phylogenetic structure of the local clades. The results of our study indicate that it may be possible to restrict MDR-TB evolution and dissemination by prioritizing screening for INH-resistant TB strains before they become MDR-TB and by adopting measures that can limit their transmission.

## RESULTS

### Basic genetic characteristics and cluster selection.

The whole-genome sequences of the 576 M. tuberculosis clinical isolates had an average depth of 79-fold (range, 22- to 137-fold). A total of 439,139 single nucleotide polymorphisms (SNPs) were detected at 16,156 sites, with an average of 762 SNPs in each strain, compared with the H37Rv reference genome. From the genotypic resistance profiles, 230 (39.9%) isolates were pansusceptible while the other 346 (60.1%) strains were resistant to at least one anti-TB drug, including 231 (40.1%) that were MDR-TB (Table 1; see Fig. S1 in the supplemental material). The drug-resistant TB strains carried an average of 4.0 (95% confidence interval [CI], 3.8 to 4.2) resistance-conferring mutations. The ancillary information related to the strains was shown in Table S1 in the supplemental material.

**TABLE 1:** The genetic characteristics of all Tibetan clinical M. tuberculosis strains and the large drug-resistant clusters included in the study

Characteristic^*a*^	Total no. (%) of samples (N = 576)	No. (%) of strains in the largest clusters (N = 372)
Lineage		
Lineage 2	524 (91.0)	372 (100)
Lineage 3	16 (2.8)	0 (0)
Lineage 4	36 (6.2)	0 (0)
Drug-resistance profile		
Drug sensitive	230 (39.9)	131 (35.2)
Drug resistant	115 (20.0)	65 (17.5)
MDR-TB	183 (31.8)	143 (38.4)
Pre-XDR-TB/XDR-TB	48 (8.3)	33 (8.9)
Genetic clustering		
5-SNP cluster	281 (48.8)	199 (53.5)
12-SNP cluster	381 (66.1)	268 (72.0)
30-SNP cluster	480 (83.3)	337 (90.6)
50-SNP cluster	518 (89.9)	363 (97.6)
No. of 50-SNP clusters	50	11
Avg 50-SNP cluster size	10.7	33.8

a MDR-TB, multidrug-resistant tuberculosis; XDR-TB, extremely drug-resistant tuberculosis; SNP, single-nucleotide polymorphism.

To identify the transmission clusters of drug-resistant TB strains, we first calculated the pairwise genetic distances between strains. The Tibetan strains showed no clear separation but rather a gradual decrease in the distribution of the SNP differences between strains (see Fig. S2 in the supplemental material). Using 5 SNPs or 12 SNPs as thresholds, 48.8% (281/576) and 66.1% (381/576) of the strains, respectively, were genetically clustered, suggesting a high level of recent transmission. The majority (89.9%, 518/576) of the strains were separated from another strain by less than a 50-SNP distance and were confirmed as phylogenetically clustered. These genomes separated into 50 long time frame clusters that included 2 to 85 strains each.

**FIG 1:**
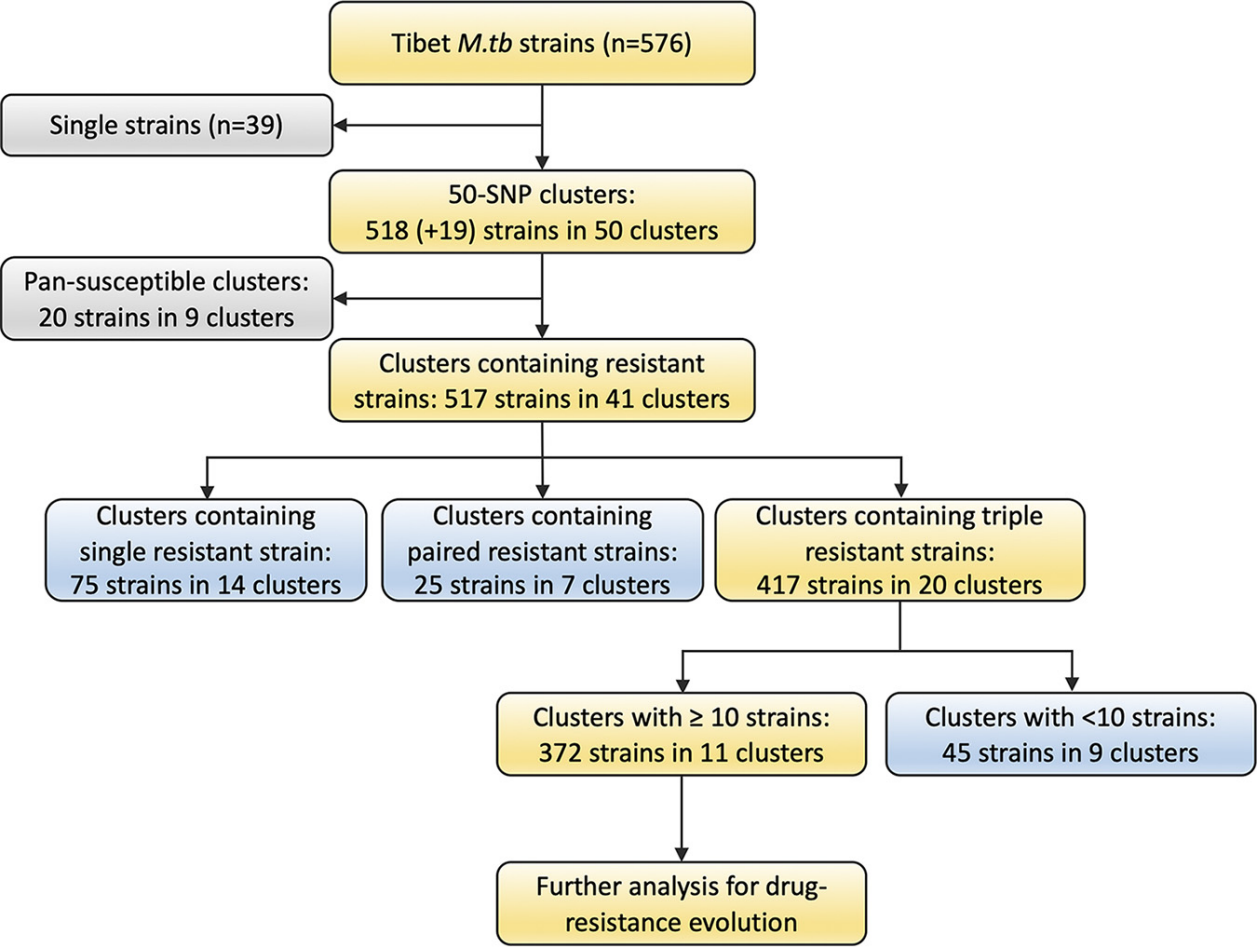
Diagram of Tibetan M. tuberculosis strains included in the study. Using a pairwise distance of 50 SNPs, most strains (89.9%, 518/576) were phylogenetically separated into 50 long-term transmission clusters. Another 19 strains were embedded in the phylogenetic clusters, although they had longer genetic distances. Finally, 11 large clusters that contained consecutive ≥3 drug-resistant strains were included for evolution analysis. The clusters included 363 50-SNP clustered strains and another 9 phylogenetically embedded strains.

**FIG 2:**
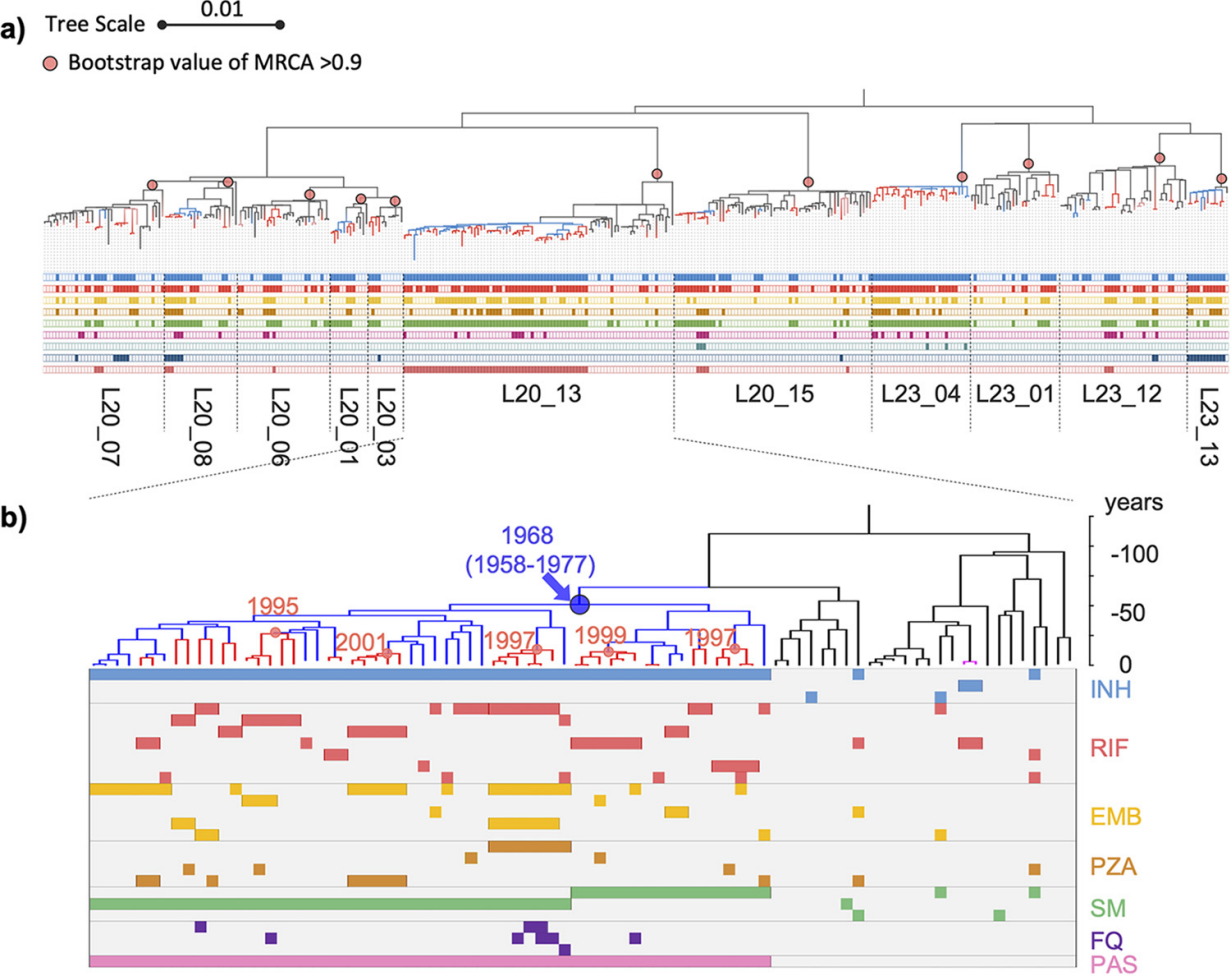
The phylogenetic tree and resistance profiles of the 11 drug-resistant clusters of tuberculosis. (a) The midpoint-rooted maximum-likelihood phylogeny tree was labeled for the 11 large genomic clusters. The pink dots indicated the most recent ancestral nodes (MRCA) of each cluster. Blue and red branches indicated isoniazid-resistance and MDR-TB, respectively. The lines below the tree indicate the presence of a resistance mutation to, from the top to bottom, isoniazid (INH), rifampicin (RIF), ethambutol (EMB), pyrazinamide (PZA), streptomycin (SM), fluoroquinolones (FQ), aminoglycosides (AG), ethionamide (ETO), and para-aminosalicylic acid (PAS). (b) The dated phylogeny of the largest cluster L20_13 was annotated with the resistance-conferring mutations, and the lines below the tree indicate the presence of mutations conferring resistance to the corresponding drugs. The labeled numbers on the tree indicate the estimated year of the nodes where the mutations for resistance to INH and RIF occurred.

To capture the process of drug-resistance evolution during the long-term transmission of M. tuberculosis, we selected the 11 largest clusters that contained at least 10 strains and at least 3 phylogenetically consecutive resistant strains for further analysis (Fig. 1). They included seven clusters of the ancient Beijing sublineage and four clusters of the modern Beijing sublineage. The clusters contained 372 strains, including 363 strains within the 50-SNP distance. The phylogenic structure with the corresponding drug resistance profiles is shown in Fig. 2. These strains, which all belonged to lineage 2, accounted for 64.6% (372/576) of the total isolates and 76.2% (176/231) of the MDR-TB isolates (Table 1).

### Resistance profiles and genetic markers in clusters.

**FIG 3:**
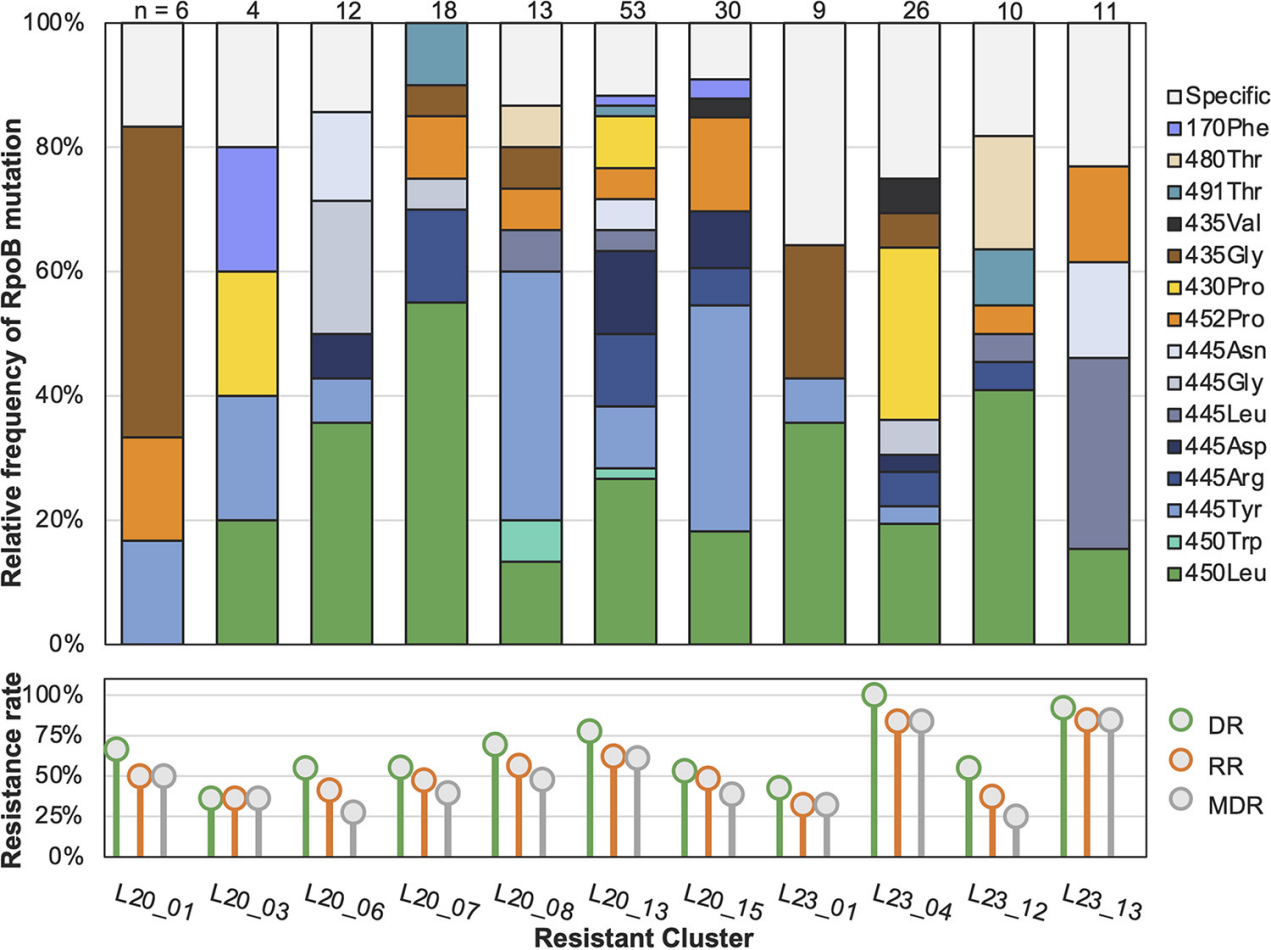
RpoB mutations in the 11 drug-resistant clusters. The clusters are shown in the order according to the phylogenetic structure in Fig. 2a. The lower subfigure shows the proportions of M. tuberculosis strains in each cluster with drug resistance to at least one drug (DR), to rifampicin (RR), or to both isoniazid and rifampicin (MDR-TB). The top subfigure shows the proportions of different RpoB mutations among rifampicin-resistant strains in each cluster. The different colors in the bars indicate the RpoB mutations shared by at least two clusters, while the sections in gray indicate a mutation that appeared in only one cluster. On the right side are the codon numbers of the mutated amino acids in the M. tuberculosis RpoB, followed by the mutant amino acids.

The proportion of rifampicin-resistant (RR) strains within the enrolled clusters ranged from 32.1% to 84.6%, and the proportions MDR-TB strains ranged from 25.0% to 83.9%. Among the 197 RR-TB strains, the most frequent RIF resistant mutations were RpoB Ser450Leu (*n* = 64, 32.5%), His445Tyr (29, 14.7%), Leu430Pro (16, 8.1%), His445Arg (15, 7.6%), Leu452Pro (15, 7.6%), His445Asp (13, 6.6%), and Asp435Gly (10, 5.1%), but the mix of these mutations differed in each cluster (Fig. 3). The clusters also had different resistance profiles and varied resistance-conferring mutations, suggesting distinct evolutionary pathways.

A total of 25 different RIF-resistance mutations were identified in the clusters, of which 15 were found in at least 2 clusters, while the others were present in just one cluster (see Table S2 in the supplemental material). On average, the clusters carried 7.6 (range, 4 to 16) different RIF-resistance mutations. The number of different RpoB mutations was correlated with the number of RIF-resistant strains in each cluster, with an *R*^2^ coefficient of 0.8883. The correlation was even stronger when we compared the number of different RpoB mutations with the number of drug-resistant (*R*^2^ = 0.9120) or INH-resistant strains (*R*^2^ = 0.9026) (see Fig. S3 in the supplemental material) in each cluster. Compensatory mutations in *rpoABC* genes were detected in 48 rifampicin-resistant strains belonging to 10 of the 11 clusters. Most compensatory mutations were present in only a single strain or in small subclusters of 2 to 3 strains, but the RpoB Gln409Arg mutation was found in a four-strain subcluster of cluster L23_12, and RpoC Asn416Ser and RpoA Gly31Ser were found in six-strain subclusters of clusters L20_07 and L20_13, respectively (Table S2).

### Transmitted and acquired resistance.

**FIG 4:**
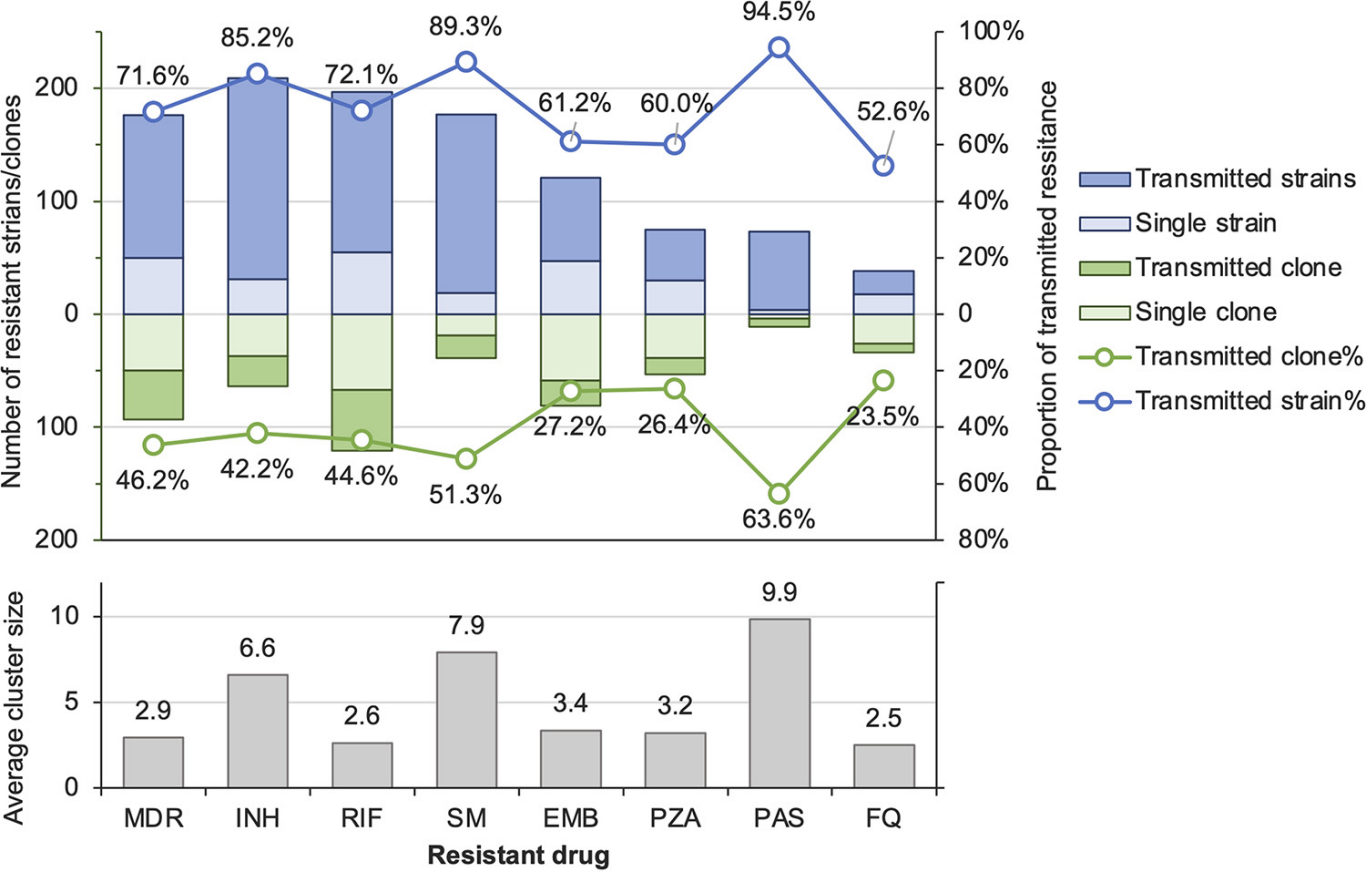
Transmitted resistance to each antituberculosis drug. The light-blue and dark-blue bars indicate the number of strains with acquired and transmitted resistance, respectively, to the drugs shown on the bottom of the figure. If the genetically clustered strains share the same resistance-conferring mutation, they are regarded as a clone resistant to the corresponding drug. The number of clones with transmitted resistance are shown in dark-green bars, while single mutants, considered acquired resistance, are shown in light green. The average sizes of transmission clusters with resistance to each indicated drug are shown in the lower subfigure.

By analyzing the nodes in the phylogenetic tree where the resistance mutations were inferred to have occurred, we found that the evolution of resistance mutations could be divided into either transmitted or acquired resistance. Transmitted resistance is when the mutation occurred on the internal nodes such that adjacent strains carry the same mutation. In contrast, acquired resistance is when the mutation is present only in a single strain on a terminal branch tip. The proportions of transmitted resistance were highest among strains resistant to para-aminosalisylic acid (PAS) (94.5% [69/73]), followed by strains resistant to streptomycin (SM) (89.3% [158/177]) and INH (85.2% [178/209]). These resistant strains evolved from 7, 20, and 27 ancestral mutant clones, respectively, and the average transmitted cluster sizes were 9.9, 7.9, and 6.6 strains, respectively (Fig. 4). The proportions of transmitted resistance among strains with resistance to RIF, ethambutol, and pyrazinamide were 72.1% (178/247), 60.7% (74/122), and 60.0% (45/75), respectively, and the sizes of the transmitted resistant clusters were generally smaller than the transmitted clusters of INH-resistant strains (2.6 to 3.4 versus 6.6). Similarly, while the majority of MDR-TB strains (71.6% [126/176]) were also transmitted from ancestral clones, they were in relatively small clusters with an average size of 2.9.

### Pathways of multidrug resistance evolution.

**FIG 5:**
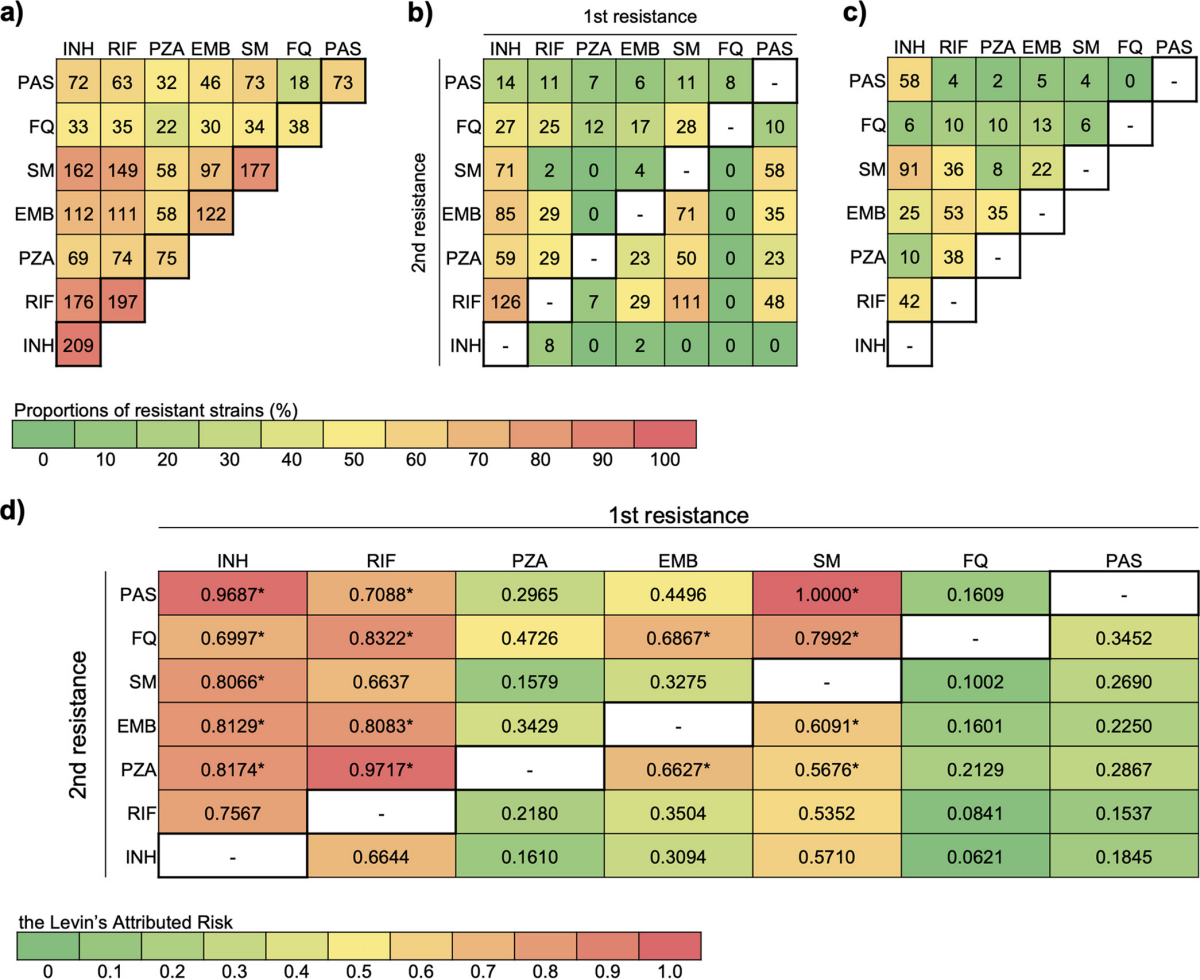
Stepwise accumulation of paired resistance in Tibetan M. tuberculosis isolates. Numbers of strains with coresistance (a), stepwise coresistance (b), and concurrent coresistance (c) are shown at the top, with the colors of the cells indicating the proportions of strains with coresistance among all the samples. Levin’s attributed risk of the order of occurrence of paired resistance is shown in d, where asterisks denote the that the risk of the indicated order was significantly higher (*P* < 0.05) than the risk of the opposite order of resistance occurrence.

We observed that mutations conferring resistance to PAS, SM, and INH occurred before resistance to other drugs, which likely explains their larger clusters and higher rates of transmitted resistance. To trace the evolution of resistance to pairs of drugs, we counted the events where resistance to drug A occurred before resistance to drug B and vice versa (Fig. 5). INH resistance overwhelmingly evolved before all other drug resistances. Among the 176 MDR-TB strains, 126 (71.6%) strains were first resistant to INH and then subsequently resistant to RIF (Fig. 5b). Only 8 (4.5%) MDR-TB strains acquired RIF resistance before INH resistance. The other 42 strains appear to have acquired resistance to both drugs at about the same time because it was impossible to determine the order in which resistance developed (Fig. 5c).

We used the method proposed by Muzondiwa et al. (8) to assess the risk of different orders of paired resistance (Fig. 5d). The correlations between resistance to paired anti-TB drugs were all strong with a high linkage disequilibrium (LD) over 0.70. Based on Levin’s attributed risk, the risk of an INH-resistant mutant accumulating resistance to other drugs was greater than the opposite order.

Except for two strains that acquired ethambutol resistance before INH resistance and eight MDR strains that first developed RIF resistance, no other drug resistance occurred before INH resistance in the strains resistant to multiple drugs. Of the strains that were resistant to INH and PAS (80.6% [58/72]) or INH and SM (56.2% [91/162]), most appeared to have acquired resistance to the two drugs at about the same time.

**FIG 6:**
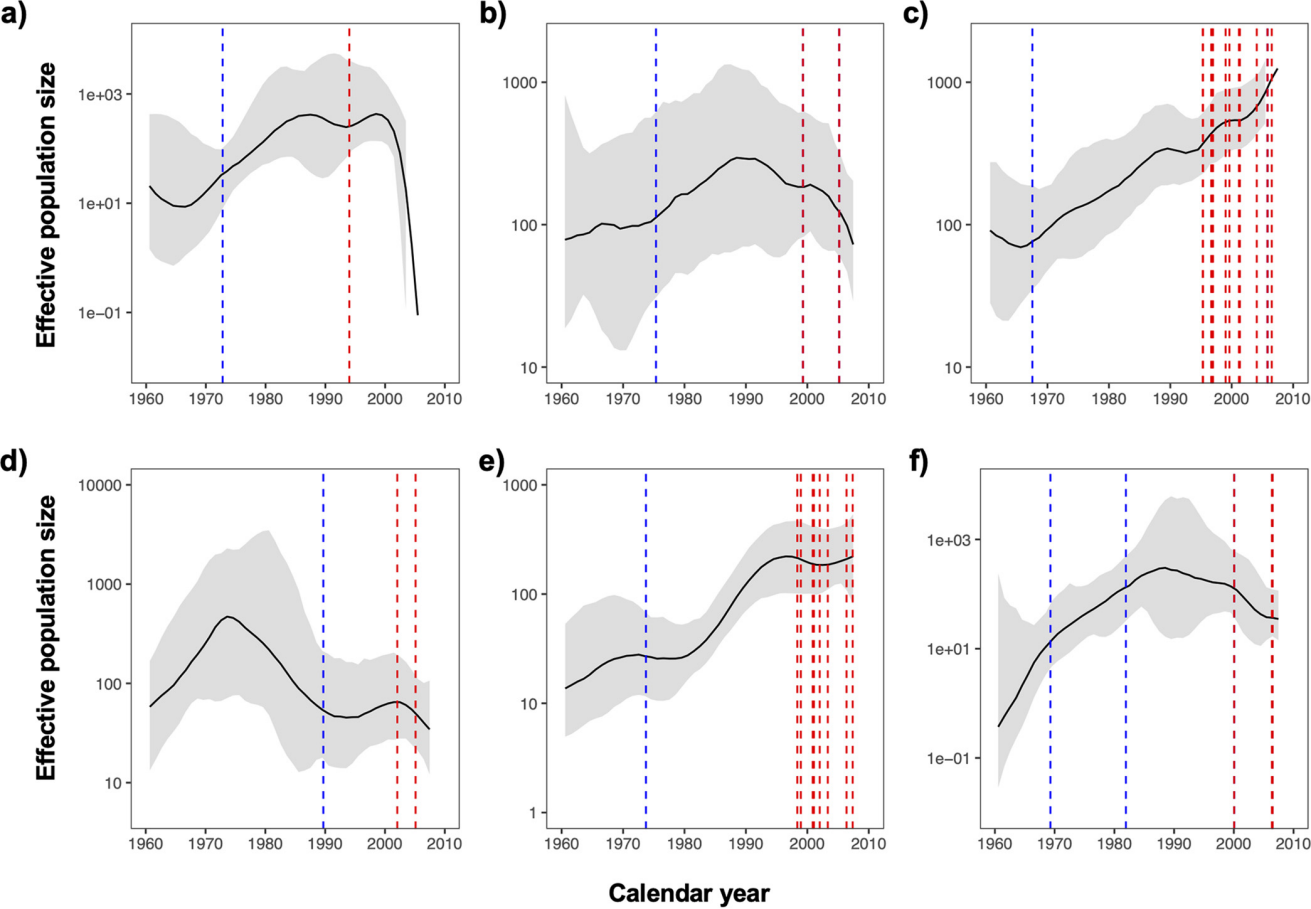
Effective population sizes of the six largest drug-resistant clusters. Blue and red dashed lines indicate the estimated acquisition dates of mutations conferring resistance to isoniazid and rifampicin, respectively. Multiple lines indicate the acquisition of mutations on different branches or second mutations conferring resistance to the same drug. Subfigures a to f corresponded to clusters L20_01, L20_08, L20_13, L20_15, L23_04, and L23_13.

To assess M. tuberculosis transmission by year in relation to resistance acquisition, we used the Bayesian skyline plots to reconstruct the expansion of the bacterial populations in the six biggest clusters (Fig. 6) and then mapped the estimated dates of where resistance to INH and RIF evolved onto the nodes. From these plots, it appears that the strains acquired INH resistance in the 1970s, which was followed by a period of exponential growth of their bacterial populations. By the late 1990s, however, when the clusters had developed additional resistance to RIF, the bacterial populations decreased in all except the largest cluster, Cluster L20_13, which had the longest time span with accumulated RIF resistance (Fig. 2b).

## DISCUSSION

In this study of clinical M. tuberculosis isolates from Tibet, China, we characterized the evolutionary trajectories of drug resistance and demonstrated that INH resistance evolved in the 1970s, before other drug resistance, and was followed by a rapid expansion of the INH-resistant bacterial population. During the 1970s to 1990s, before RIF was widely used in Tibet, the INH-resistant strains were unlikely to be cured with the triple-drug therapy used at the time—INH, PAS, and streptomycin. This 20-year interval allowed the extensive transmission of INH-resistant strains to form large clusters with a high risk of developing additional resistance. By the late 1990s, when RIF was introduced into the primary four-drug treatment regimen, these INH-resistant clusters began to accumulate a variety of mutations conferring resistance to RIF and became MDR-TB. This phylogenetic analysis demonstrates that the current high rate of MDR-TB in Tibet was driven by the emergence and the expansion of these INH-resistant M. tuberculosis strains.

Because Tibet is relatively isolated, we could previously document the local adaptation of M. tuberculosis strains (4), and here, we could trace the evolution and transmission of drug-resistant tuberculosis over a fairly long time frame. We estimated that the proportion of transmitted resistance among MDR-TB was 71.6%, which is higher than the value calculated using pairwise SNP distances of 5 or 12 SNPs. These lower SNP thresholds are often used to define cases arising from direct or recent transmission (9, 10), but they will not capture the evolution and spread of drug-resistant M. tuberculosis strains over a longer time period. For example, a study from South Africa traced the long-term spread of an extensively drug-resistant TB (XDR-TB) strain into a highly monophyletic clade of strains with an average pairwise genetic distance of 21.07 SNPs, ranging from 0 to 104 SNPs (6).

A recent study, similar to ours, traced the cross-continental expansion of the modern Beijing lineage W148 clade, whose members differed by a median of 32 SNPs. After nearly 30 years of initial INH resistance, RIF resistance emerged in the W148 clade around 1991, with at least 66 independent RIF resistance events, followed by the subsequent development of XDR and pre-XDR strains (7, 11). The acquisition of INH resistance prior to RIF resistance has also been demonstrated in other successful MDR clades (12, 13), most often with the KatG Ser315Thr mutation that confers high-level INH resistance with minimal fitness costs (14). *In vitro* experiments have shown that M. tuberculosis spontaneously acquires INH resistance mutations more frequently than RIF resistance mutations (15).

Although it was thought that INH resistance could be cured routinely with the standard first-line therapy, a meta-analysis found high proportions of unfavorable outcomes among INH-resistant TB patients treated with the first-line regimen of INH, RIF, pyrazinamide, and ethambutol (HRZE), with frequencies of failure, relapse, and acquired multidrug resistance of 11%, 10%, and 8%, respectively (16). Worldwide, INH-resistant strains were found in 7.4% of new and 11.4% of retreated TB patients (17). Improved detection and effective treatment of these INH-resistant strains could help restrict the future evolution of MDR-TB.

This study had some limitations. We selected only the major clusters to illustrate the evolutionary pathways of drug resistance over a long time frame, and the resistance rates and the proportions of resistance transmission may be different if the analysis included the whole data set or all TB in Tibet. In addition, the passive case finding and limited diagnosis capacity in Tibet during the sampling period may have missed a substantial, but unknown proportion of incident TB. As a result, although we found high proportions of clustering and inferred the transmission of drug-resistant strains, it is likely that these data underestimate the true rates of clustering and MDR-TB transmission in Tibet.

In conclusion, the M. tuberculosis clusters emerged and expanded after acquiring INH resistance and became the precursors of the current MDR-TB burden in Tibet. Early detection of INH-resistant TB and full drug resistance profiles are essential to avoid further amplification and transmission of drug resistance.

## MATERIALS AND METHODS

### Study site and sampling.

Tibet is located on the world’s highest plateau, with an average altitude of over 4,000 meters above sea level. Its large expanse of 1.2 million square kilometers constitutes nearly an eighth of China’s entire landmass but contains only 3.3 million inhabitants (<3 people per square kilometer). In a previous epidemiological study (18), M. tuberculosis isolates were collected from 576 patients presenting to 7 municipal-level clinics in Tibet during the years 2006, 2009, and 2010. Smear-positive patients were sequentially enrolled, and their treatment-naive sputum specimens were sent to the municipal central laboratory for culturing. The 576 isolates that were available and included in the study represent an estimated 40% of the smear-positive pulmonary TB patients diagnosed during the three sampling years. Only one strain per patient was included in our data set, except for second isolates from patients beginning a retreatment regimen. These 576 isolates were whole-genome sequenced, the genotyping showed that the majority belonged to lineage 2, and isolates were separated into several highly monophyletic clades (4). In this study, we selected the drug-resistant clades to investigate the emergence of resistance and the stepwise evolution into multidrug resistance.

### Whole-genome sequencing and SNP calling.

Genomic DNA of the M. tuberculosis isolates was extracted with the cetyltrimethylammonium bromide method (19). A 300-base-pair double-ended DNA library of each isolate was sequenced on the Illumina HiSeq 2500 platform with an expected depth of 100-fold. After the removal of low-quality reads with Trimmomatic (v0.39), the reads were aligned to the H37Rv reference genome (accession no. AL123456.3) with BWA-MEM (v0.6) and single nucleotide polymorphisms (SNPs) were called with the GATK tool (HaplotypeCaller v4.1.4.1). SNPs in the repetitive regions (e.g., PPE/PE-PGRS family genes) and in drug-resistant genes were removed with VCFtools. Confidential fixed mutations with a frequency of ≥75% and depth of ≥10 were kept and converted into a FASTA-format alignment for phylogenic reconstruction.

Genetic distance was calculated by comparing the sequences of each pair of strains to generate an SNP matrix. Genomic clusters were identified using different thresholds of pairwise SNP distances. Strains separated by 5 or 12 SNPs were considered “close clusters” that emerged over short-term windows of several years (9). Strains within a 50-SNP distance were considered “remote clusters” that formed over several decades that include the entire era of anti-TB chemotherapy beginning in the 1950s (20). The sequencing data were deposited in the Sequence Read Archive of the National Center for Biotechnology Information (accession no. PRJNA656167).

### Phylogeny construction and phylodynamic analysis.

The identified SNPs were used to construct a phylogeny tree with RAxML software (21), employing the maximum-likelihood method with a general time-reversible model of nucleotide substitution and main node values over 0.7 after 500 bootstrapping trees. The aligned sequences of the major drug-resistant clades were also analyzed with BEAST 2 software (v2.6.1) (22) to construct a dated phylogeny based on a molecular clock with an uncorrelated log-normal distribution and a tree generated prior with coalescent Bayesian skyline analysis. The molecular clock was set at 1.14 (0.49 to 1.80) × 10^−7^ substitutions per site per year (0.50 [0.22 to 0.79] SNPs per genome·per year), which is a mutation rate reported elsewhere for lineage 2 strains (23). Bayesian skyline plots for the effective population size were reconstructed in Tracer software (24), with the age of the most recent common ancestor (MRCA) node being the height of the tree. The phylogenies were visualized with iTOL (v6; https://itol.embl.de/).

### Determination of transmitted and acquired genotypic resistance.

Known drug resistance mutations with a frequency of ≥5% in the sequence alignments were identified using the TB-profiler tool (v4.4.0) (25), based on a published database of mutations conferring resistance to 14 anti-TB drugs (26). Compensatory mutations were identified based on a previously reported database (27). Transmitted and acquired resistance were determined by mapping the resistance mutations onto a phylogenetic tree (5) using the following rules: mutations shared by all strains on a branch were considered to be present at the MRCA of the branch and termed “transmitted resistance,” and these strains were considered to be phylogenetically clustered; and mutations on single branches and not present in neighboring branches were assumed to have occurred at the terminal tip and were considered “acquired resistance.”

### Ethics approval and consent to participate.

Ethical approval was obtained from the Ethical Review Board of the Chinese Center for Disease Control and Prevention (no. ICDC-2019010).

### Data availability.

Sequencing reads have been submitted to the NCBI or The European Bioinformatics Institute under study accession number PRJNA656167. In-house scripts developed by Phelan et al. (25) were used to automate the processing of raw sequencing reads, to generate an SNP distance matrix, and to identify genetic markers for drug resistance. The scripts have been deposited previously at GitHub (https://github.com/pathogenseq/fastq2matrix; https://github.com/jodyphelan/TBProfiler).

## References

[1] World Health Organization. 2021. Global tuberculosis report 2021. World Health Organization, Geneva, Switzerland.

[2] Li B, Zhang X, Guo J, Wang J, Pianduo B, Wei X, Yin T, Hu J. 2019. Prevalence of pulmonary tuberculosis in Tibet Autonomous Region, China, 2014. Int J Tuber Lung Dis 23:735–740. doi:10.5588/ijtld.18.0614. 31315707

[3] Yang J, Yangla Bassan-Junda Li C, Du B. 2014. Analysis on the drug resistance of Mycobacterium tuberculosis in Lhasa area, Tibet. J Tibet Univ (in Chinese) 29:36–39.

[4] Liu Q, Liu H, Shi L, Gan M, Zhao X, Lyu LD, Takiff HE, Wan K, Gao Q. 2021. Local adaptation of Mycobacterium tuberculosis on the Tibetan plateau. Proc Natl Acad Sci USA 118:e2017831118. doi:10.1073/pnas.2017831118. 33879609 PMC8092575

[5] Casali N, Nikolayevskyy V, Balabanova Y, Harris SR, Ignatyeva O, Kontsevaya I, Corander J, Bryant J, Parkhill J, Nejentsev S, Horstmann RD, Brown T, Drobniewski F. 2014. Evolution and transmission of drug-resistant tuberculosis in a Russian population. Nat Genet 46:279–286. doi:10.1038/ng.2878. 24464101 PMC3939361

[6] Brown TS, Challagundla L, Baugh EH, Omar SV, Mustaev A, Auld SC, Shah NS, Kreiswirth BN, Brust JCM, Nelson KN, Narechania A, Kurepina N, Mlisana K, Bonneau R, Eldholm V, Ismail N, Kolokotronis S-O, Robinson DA, Gandhi NR, Mathema B. 2019. Pre-detection history of extensively drug-resistant tuberculosis in KwaZulu-Natal, South Africa. Proc Natl Acad Sci USA 116:23284–23291. doi:10.1073/pnas.1906636116. 31659018 PMC6859317

[7] Merker M, Rasigade J-P, Barbier M, Cox H, Feuerriegel S, Kohl TA, Shitikov E, Klaos K, Gaudin C, Antoine R, Diel R, Borrell S, Gagneux S, Nikolayevskyy V, Andres S, Crudu V, Supply P, Niemann S, Wirth T. 2022. Transcontinental spread and evolution of Mycobacterium tuberculosis W148 European/Russian clade toward extensively drug resistant tuberculosis. Nat Commun 13:5105. doi:10.1038/s41467-022-32455-1. 36042200 PMC9426364

[8] Muzondiwa D, Hlanze H, Reva ON. 2021. The epistatic landscape of antibiotic resistance of different clades of mycobacterium tuberculosis. Antibiotics 10:857. doi:10.3390/antibiotics10070857. 34356778 PMC8300818

[9] Walker TM, Ip CLC, Harrell RH, Evans JT, Kapatai G, Dedicoat MJ, Eyre DW, Wilson DJ, Hawkey PM, Crook DW, Parkhill J, Harris D, Walker AS, Bowden R, Monk P, Smith EG, Peto TEA. 2013. Whole-genome sequencing to delineate Mycobacterium tuberculosis outbreaks: a retrospective observational study. Lancet Infect Dis 13:137–146. doi:10.1016/S1473-3099(12)70277-3. 23158499 PMC3556524

[10] Yang C, Luo T, Shen X, Wu J, Gan M, Xu P, Wu Z, Lin S, Tian J, Liu Q, Yuan Z, Mei J, DeRiemer K, Gao Q. 2017. Transmission of multidrug-resistant Mycobacterium tuberculosis in Shanghai, China: a retrospective observational study using whole-genome sequencing and epidemiological investigation. Lancet Infect Dis 17:275–284. doi:10.1016/S1473-3099(16)30418-2. 27919643 PMC5330813

[11] Merker M, Blin C, Mona S, Duforet-Frebourg N, Lecher S, Willery E, Blum MGB, Rüsch-Gerdes S, Mokrousov I, Aleksic E, Allix-Béguec C, Antierens A, Augustynowicz-Kopeć E, Ballif M, Barletta F, Beck HP, Barry CE, Bonnet M, Borroni E, Campos-Herrero I, Cirillo D, Cox H, Crowe S, Crudu V, Diel R, Drobniewski F, Fauville-Dufaux M, Gagneux S, Ghebremichael S, Hanekom M, Hoffner S, Jiao W-w, Kalon S, Kohl TA, Kontsevaya I, Lillebæk T, Maeda S, Nikolayevskyy V, Rasmussen M, Rastogi N, Samper S, Sanchez-Padilla E, Savic B, Shamputa IC, Shen A, Sng L-H, Stakenas P, Toit K, Varaine F, Vukovic D, et al. 2015. Evolutionary history and global spread of the Mycobacterium tuberculosis Beijing lineage. Nat Genet 47:242–249. doi:10.1038/ng.3195. 25599400 PMC11044984

[12] Eldholm V, Monteserin J, Rieux A, Lopez B, Sobkowiak B, Ritacco V, Balloux F. 2015. Four decades of transmission of a multidrug-resistant Mycobacterium tuberculosis outbreak strain. Nat Commun 6:7119. doi:10.1038/ncomms8119. 25960343 PMC4432642

[13] Casali N, Broda A, Harris SR, Parkhill J, Brown T, Drobniewski F. 2016. Whole genome sequence analysis of a large isoniazid-resistant tuberculosis outbreak in London: a retrospective observational study. PLoS Med 13:e1002137. doi:10.1371/journal.pmed.1002137. 27701423 PMC5049847

[14] Manson AL, Cohen KA, Abeel T, Desjardins CA, Armstrong DT, Barry CE, Brand J, Chapman SB, Cho S-N, Gabrielian A, Gomez J, Jodals AM, Joloba M, Jureen P, Lee JS, Malinga L, Maiga M, Nordenberg D, Noroc E, Romancenco E, Salazar A, Ssengooba W, Velayati AA, Winglee K, Zalutskaya A, Via LE, Cassell GH, Dorman SE, Ellner J, Farnia P, Galagan JE, Rosenthal A, Crudu V, Homorodean D, Hsueh P-R, Narayanan S, Pym AS, Skrahina A, Swaminathan S, Van der Walt M, Alland D, Bishai WR, Cohen T, Hoffner S, Birren BW, Earl AM, III, TBResist Global Genome Consortium. 2017. Genomic analysis of globally diverse Mycobacterium tuberculosis strains provides insights into the emergence and spread of multidrug resistance. Nat Genet 49:395–402. doi:10.1038/ng.3767. 28092681 PMC5402762

[15] Ford CB, Shah RR, Maeda MK, Gagneux S, Murray MB, Cohen T, Johnston JC, Gardy J, Lipsitch M, Fortune SM. 2013. Mycobacterium tuberculosis mutation rate estimates from different lineages predict substantial differences in the emergence of drug-resistant tuberculosis. Nat Genet 45:784–790. doi:10.1038/ng.2656. 23749189 PMC3777616

[16] Gegia M, Winters N, Benedetti A, van Soolingen D, Menzies D. 2017. Treatment of isoniazid-resistant tuberculosis with first-line drugs: a systematic review and meta-analysis. Lancet Infect Dis 17:223–234. doi:10.1016/S1473-3099(16)30407-8. 27865891

[17] Dean AS, Zignol M, Cabibbe AM, Falzon D, Glaziou P, Cirillo DM, Köser CU, Gonzalez-Angulo LY, Tosas-Auget O, Ismail N, Tahseen S, Ama MCG, Skrahina A, Alikhanova N, Kamal SMM, Floyd K. 2020. Prevalence and genetic profiles of isoniazid resistance in tuberculosis patients: a multicountry analysis of cross-sectional data. PLoS Med 17:e1003008. doi:10.1371/journal.pmed.1003008. 31961877 PMC6974034

[18] Dong H, Shi L, Zhao X, Sang B, Lv B, Liu Z, Wan K. 2012. Genetic diversity of Mycobacterium tuberculosis isolates from Tibetans in Tibet, China. PLoS One 7:e33904. doi:10.1371/journal.pone.0033904. 22479472 PMC3316506

[19] Schiebelhut LM, Abboud SS, Gomez Daglio LE, Swift HF, Dawson MN. 2017. A comparison of DNA extraction methods for high-throughput DNA analyses. Mol Ecol Resour 17:721–729. doi:10.1111/1755-0998.12620. 27768245

[20] Stimson J, Gardy J, Mathema B, Crudu V, Cohen T, Colijn C. 2019. Beyond the SNP threshold: identifying outbreak clusters using inferred transmissions. Mol Biol Evol 36:587–603. doi:10.1093/molbev/msy242. 30690464 PMC6389316

[21] Stamatakis A. 2014. RAxML version 8: a tool for phylogenetic analysis and post-analysis of large phylogenies. Bioinformatics 30:1312–1313. doi:10.1093/bioinformatics/btu033. 24451623 PMC3998144

[22] Bouckaert R, Vaughan TG, Barido-Sottani J, Duchêne S, Fourment M, Gavryushkina A, Heled J, Jones G, Kühnert D, De Maio N, Matschiner M, Mendes FK, Müller NF, Ogilvie HA, Du Plessis L, Popinga A, Rambaut A, Rasmussen D, Siveroni I, Suchard MA, Wu C-H, Xie D, Zhang C, Stadler T, Drummond AJ. 2019. BEAST 2.5: an advanced software platform for Bayesian evolutionary analysis. PLoS Comput Biol 15:e1006650. doi:10.1371/journal.pcbi.1006650. 30958812 PMC6472827

[23] Menardo F, Duchene S, Brites D, Gagneux S. 2019. The molecular clock of Mycobacterium tuberculosis. PLoS Pathog 15:e1008067. doi:10.1371/journal.ppat.1008067. 31513651 PMC6759198

[24] Rambaut A, Drummond AJ, Xie D, Baele G, Suchard MA. 2018. Posterior summarization in Bayesian phylogenetics using Tracer 1.7. Syst Biol 67:901–904. doi:10.1093/sysbio/syy032. 29718447 PMC6101584

[25] Phelan JE, O'Sullivan DM, Machado D, Ramos J, Oppong YEA, Campino S, O'Grady J, McNerney R, Hibberd ML, Viveiros M, Huggett JF, Clark TG. 2019. Integrating informatics tools and portable sequencing technology for rapid detection of resistance to anti-tuberculous drugs. Genome Med 11:41. doi:10.1186/s13073-019-0650-x. 31234910 PMC6591855

[26] Coll F, Phelan J, Hill-Cawthorne GA, Nair MB, Mallard K, Ali S, Abdallah AM, Alghamdi S, Alsomali M, Ahmed AO, Portelli S, Oppong Y, Alves A, Bessa TB, Campino S, Caws M, Chatterjee A, Crampin AC, Dheda K, Furnham N, Glynn JR, Grandjean L, Minh Ha D, Hasan R, Hasan Z, Hibberd ML, Joloba M, Jones-López EC, Matsumoto T, Miranda A, Moore DJ, Mocillo N, Panaiotov S, Parkhill J, Penha C, Perdigão J, Portugal I, Rchiad Z, Robledo J, Sheen P, Shesha NT, Sirgel FA, Sola C, Oliveira Sousa E, Streicher EM, Helden PV, Viveiros M, Warren RM, McNerney R, Pain A, et al. 2018. Genome-wide analysis of multi- and extensively drug-resistant Mycobacterium tuberculosis. Nat Genet 50:307–316. doi:10.1038/s41588-017-0029-0. 29358649

[27] Gygli SM, Loiseau C, Jugheli L, Adamia N, Trauner A, Reinhard M, Ross A, Borrell S, Aspindzelashvili R, Maghradze N, Reither K, Beisel C, Tukvadze N, Avaliani Z, Gagneux S. 2021. Prisons as ecological drivers of fitness-compensated multidrug-resistant Mycobacterium tuberculosis. Nat Med 27:1171–1177. doi:10.1038/s41591-021-01358-x. 34031604 PMC9400913

