# Supplementary material for: The Evolution and Transmission Dynamics of Multidrug-Resistant Tuberculosis in an Isolated High-Plateau Population of Tibet, China
Source: Microbiol Spectr. 2023 Mar 13;11(2):e03991-22. doi: 10.1128/spectrum.03991-22 (PMC10101056; doi:10.1128/spectrum.03991-22)
Supplement: Supplemental file 1 — Supplemental material. Download spectrum.03991-22-s0001.pdf, PDF file, 0.5 MB [file spectrum.03991-22-s0001.pdf]

Supplementary tables and figures for the manuscript "The evolution and transmission dynamics of multidrug-resistant tuberculosis in an isolated high-plateau population" by Qi Jiang, et al.

Suppl. Table 2. Rifampicin resistance RpoB mutations and compensatory mutations in the 11 largest drug-resistant *M. tuberculosis* clusters in Tibet.

Cluster ID	Total	RR	Shared mutation in RopB	Unique mutation	Compensatory mutation
L20_01	12	6	445Tyr(1), 452Pro(1), 435Gly(3)	445Ser(1)	-
L20_03	11	4	450Leu(1), 445Tyr(1), 430Pro(1), 170Phe(1)	435Ala(1)	RpoC_484Gly(1), RpoC_517Leu(1)
L20_06	29	12	450Leu(1), 445Tyr(1)/Asp(1)/Gly(3)	483Leu(1), 400Ala(1)	RpoC_698Ser(1), RpoC_484Gly(2)
L20_07	38	18	450Leu(11), 445Arg(3)/Gly(1), 452Pro(2), 435Gly(1), 491Thr(2)	-	RpoA_187Ala(1), RpoC_483Gly(2), RpoC_416Ser(6)
L20_08	23	13	450Leu(2)/Trp(1), 445Tyr(6)/Leu(1), 452Pro(1), 435Gly(1), 480Thr(1)	491Phe(2)	RpoA_181Ala(2)
L20_13	85	53	450Leu(16)/Trp(1), 445Tyr(6)/Arg(7)/Asp(8)/Leu(2), 452Pro(3), 430Pro(5), 491Thr(1), 170Phe(1)	445Pro(1), 437Asp(2), 437Thr(1), 441Leu(1), 493Leu(2)	RpoC_483Gly(2)/Ala(1), RpoC_332Ser(1) RpoA_31Ser(6), RpoC_698Ser(2), RpoC_491Thr(1)
L20_15	62	30	450Leu(6), 445Tyr(12)/Arg(2)/Asp(3), 452Pro(5), 435Val(1), 170Phe(1)	429His(2), 289_290insTGTCGT(1)	RpoC_491Val(1)
L23_01	28	9	450Leu(5), 445Tyr(1), 435Gly(3)	450Cys(3), 452Ser(2)	RpoA_187Ala(1)
L23_04	31	26	450Leu(7), 445Tyr(1)/Arg(2)/Asp(1), 430Pro(10), 435Gly(2)/Val(2)	433Leu(2), 431Gly(3), 435Tyr(4)	RpoC_1252Leu(1), RpoC_483Gly(1), RpoB_451Val(1), RpoC_433Ser(3), RpoA_183Gly(1)
L23_12	40	15	450Leu(9), 445Arg(1)/Leu(1), 452Pro(1), 491Thr(2), 480Thr(4)	432Glu(3), 435Phe(1)	RpoB_409Arg(4), RpoC_516Pro(1)
L23_13	13	11	450Leu(2), 445Leu(4), 452Pro(2)	1282_1290del(3)	RpoC_1040Arg(1), RpoC_517Leu(3), RpoC_483Ala(1)

Note: RR, Rifampicin-resistant; mutations were expressed as the *M. tuberculosis* RpoB codon number followed by the mutant amino acid, with the numbers of strains with this mutation in brackets. Unique mutations refer to mutations found in only one cluster.

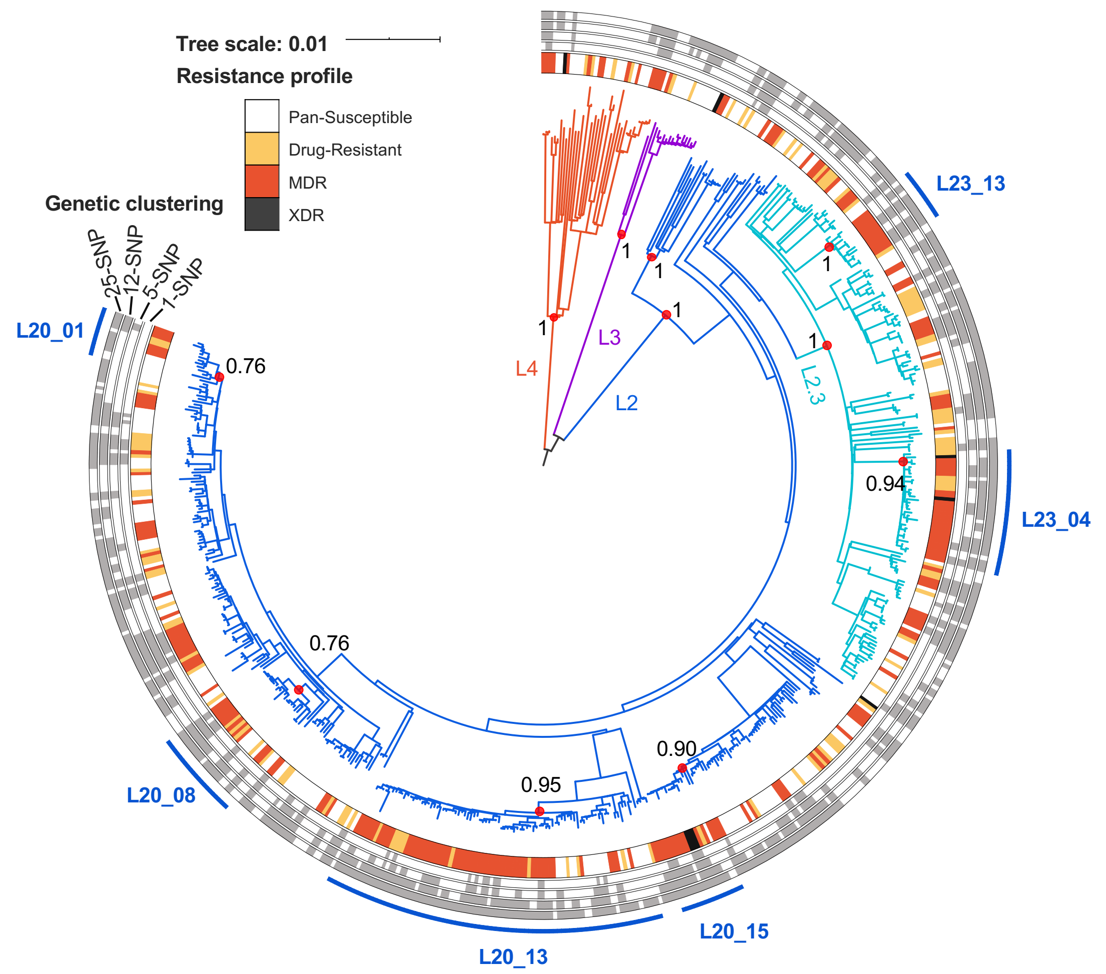

Suppl. Figure 1. Phylogenetic tree of the 576 Tibetan strains, annotated with clustering and resistance profiles. The maximum-likelihood phylogeny tree of 576 *M. tuberculosis* clinical isolates sampled during years 2006 and 2009-2010 in Tibet was rooted at the midpoint with key nodes gaining a bootstrap value over 0.7. Bootstrap values are shown as numbers on major nodes and the ancestral nodes for the six biggest drug-resistant clusters (labeled corresponding to Figure 1). The innermost surrounding circle indicates the genotypic resistance profile of each strain, including pan-susceptible (white), drug resistant (yellow), multidrug-resistant (MDR) (orange), and extremely drug-resistant (XDR) *M. tuberculosis* strains (black). The outermost surrounding circles indicate genetic clustering with thresholds of 1-, 5-, 12-, and 25-SNP distances.

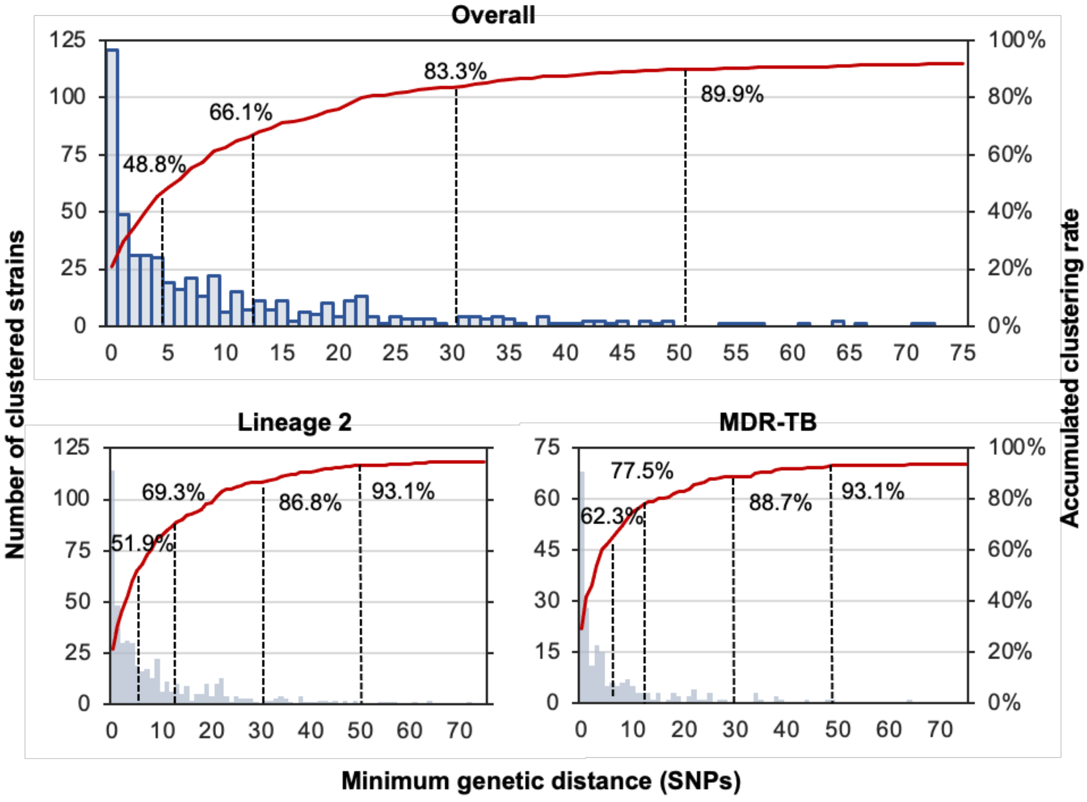

Suppl. Figure 2. Distribution of minimum genetic distance and accumulated clustering rates overall, and among Lineage 2 and multidrug-resistant tuberculosis (MDR-TB) strains. Vertical lines indicate the proportions of strains in genomic clusters defined by thresholds of 5-SNP, 12-SNP, 30-SNP and 50-SNP, respectively.

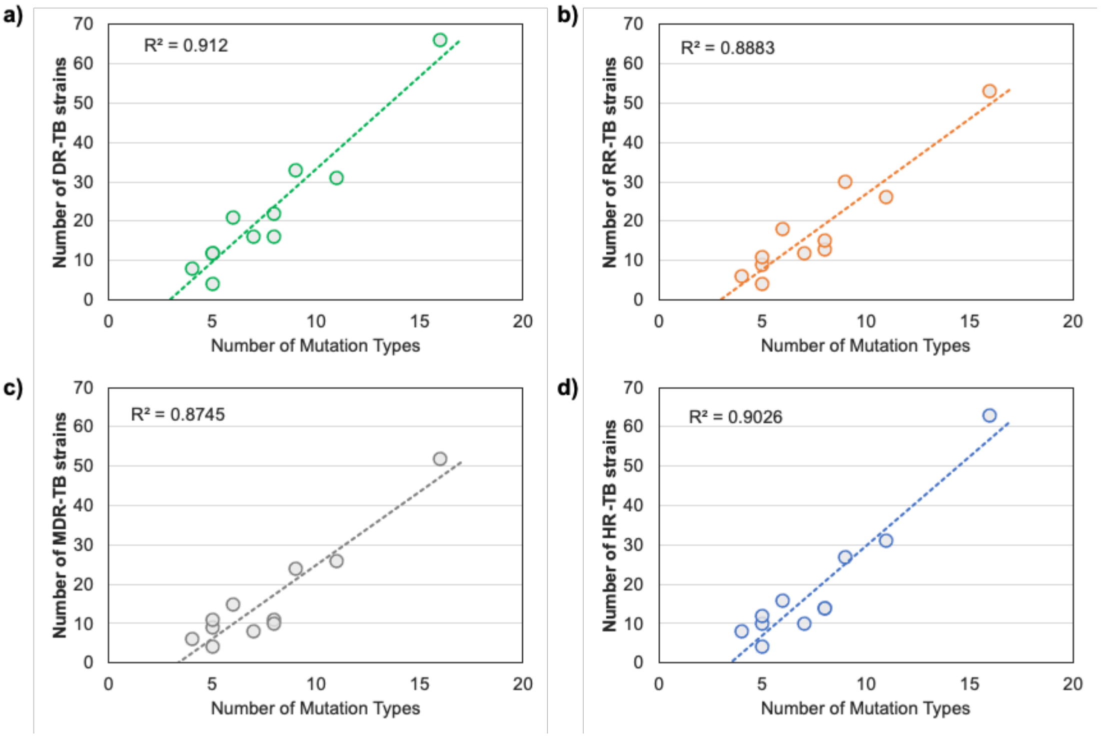

Suppl. Figure 3. Correlations between the number of mutation types and the number of drug-resistant strains among clusters of: (a), Drug-Resistant (DR); (b), Rifampicin-Resistant (RR); (c), Multidrug-Resistant (MDR) and d), Isoniazid-Resistant (HR) Tibetan strains of *M. tuberculosis*. Each dot indicates a cluster. The dashed fitted line was estimated using linear regression, with the correlation coefficient R^2 shown at the upper left corner in each graph.
